# Utility of digital images captured after 4 h of incubation on a microbiology laboratory automation system in guiding the work-up of subcultures from positive blood cultures

**DOI:** 10.1128/jcm.01320-24

**Published:** 2024-12-20

**Authors:** Melvilí Cintrón, Brenden Clark, Edwin Miranda, N. Esther Babady

**Affiliations:** 1 Clinical Microbiology Service, Department of Pathology and Laboratory Medicine, Memorial Sloan Kettering Cancer Center 5803 , New York, New York, USA; 2 Infectious Disease Service, Department of Medicine, Memorial Sloan Kettering Cancer Center 5803 , New York, New York, USA; NorthShore University Health System Department of Pathology and Laboratory Medicine, Evanston, Illinois, USA

**Keywords:** rapid identification, microbiology laboratory automation, bloodstream infections

## Abstract

Initial workup [e.g., identification (ID) and/or antimicrobial susceptibility testing (AST)] of bacterial growth on solid media traditionally occurs 16–24 h after sub-culturing of positive blood cultures (BC). Early ID and AST can be facilitated by reviewing digital images captured using a Microbiology Laboratory Automation (MLA) system. The goal of this study was to evaluate the utility of images captured at 4 h on the WASPLab MLA system for rapid bacterial ID and AST. Retrospective review of all positive BCs results between 1 January 2021 and 31 July 2022 was performed. WASPLab App data were extracted to determine the decision (e.g., perform ID and/or AST or re-incubate plates) made from the 4 h image. Culture results were extracted from the laboratory information system (LIS). A total of 6,845 BCs flagged positive during the study period. The 4 h images for 1,476 cultures (21.6%) were reviewed: 1,200 cultures were re-incubated due to insufficient growth and 276 cultures (4.0%) were sent for ID and/or AST. ID by mass spectrometry was in 100% agreement with that of the molecular BC identification panels. Overall categorical agreement between AST results from the 4 h and overnight growth was 98%. The 4 h images for the remaining 5,369 cultures (78.4%) were not available for review during the day shift. Implementing early reading times for BCs on MLA allows for rapid and accurate ID and AST results. However, optimization of the reading schedule to align with the laboratory’s operation schedule is key to realizing the full potential of early reading times.

**IMPORTANCE:**

In recent years, an increasing number of clinical microbiology laboratories have adopted laboratory automation for processing and incubation of specimens submitted for bacterial culture. At our institution, we implemented the Copan WASPLab in 2018 for all cultures, including positive blood cultures. Given that positive blood cultures start with a higher biomass of organisms, the first image capture was set up to occur after 4 h of incubation. In this study, we investigated the utility of this early 4 h image by capturing and calculating the percentage of useful actions taken based on growth identified on the image and the yield of both new identification by MALDI-TOF MS and valid and accurate antimicrobial susceptibility testing (AST) results. We found that while the 4-hour time point provided accurate, early identification and AST results, the overall yield was minimal. From a practical standpoint, this review prompted us to discontinue capture and review of this time point. While our staffing model is likely responsible for this low yield, we hope that our experience would help other laboratories decide how to implement WASPLab workflow for positive blood cultures. Thus, we believe that this information will be of interest to the readers of JCM.

## INTRODUCTION

Rapid organism identification (ID) and antimicrobial susceptibility testing (AST) in patients with bloodstream infections (BSI) are critical to implementing targeted therapy and reducing overall mortality (1). Several platforms that provide rapid organism ID and detection of common resistance markers directly from positive blood cultures (BC) are currently available. However, for most of these platforms, only a limited number of antimicrobial resistance markers are targeted for detection. Additionally, genotypic testing of these few resistance markers does not replace the need for further phenotypic AST. Previous studies have reported on the utility of Matrix-Assisted Laser Desorption Ionization-Time of Flight Mass Spectrometry (MALDI-TOF MS) directly from positive blood cultures for rapid organism ID (2–6). While an ID can be obtained in approximately 1 h after blood culture positivity, limitations of this approach include the hands-on time and manipulation required and the inadequacy of the methods for polymicrobial infections. In another approach, shorter subculture incubation (i.e., 4–6 h), instead of the standard 18–24 h incubation, was shown to yield high concordance with results obtained following standard incubation (7, 8).

The implementation of Microbiology Laboratory Automation (MLA) in clinical laboratories offers the opportunity to improve on traditional blood culture workflows. MLA allows for user-defined timing of image captures to evaluate the presence of organism growth ready for identification and additional work-up. The aim of this study was to (i) evaluate the utility of a 4 h imaging time point on the MLA to confirm the presence of sufficient growth for downstream identification and AST, (ii) to investigate the added value of MALDI-TOF MS ID on the 4 h growth compared to the BCID results, and (iii) to compare the agreement between AST patterns from the 4 h growth compared to standard 18–24 h subculture growth.

## MATERIALS AND METHODS

### Study setting

This was a retrospective study of blood cultures performed between January 2021 and July 2022 at Memorial Sloan Kettering Cancer Center (MSK), a 514-bed tertiary cancer care center in New York City. The WASPLab MLA was introduced at MSK in 2018 and all bacteriology cultures, including positive blood cultures, are routinely processed on the WASPLab. The ePlex BCID was introduced at MSK in 2019 and performed on the first positive blood culture for each new episode of bacteremia or fungemia. The laboratory is open 24 h/day and 7 days a week (24/7). Gram staining and initial ePlex identification of positive blood cultures are performed 24/7. Additional positive cultures work-up occurs only during the day shift (8 am–4 pm). The study protocol was reviewed and approved by the Institutional Review Board (IRB) of MSK and granted a waiver of informed consent.

### Blood cultures workflow

**Fig 1:**
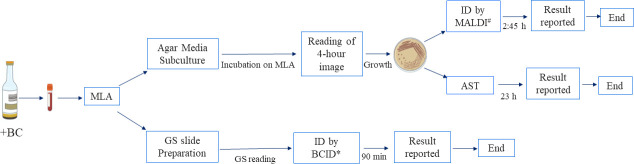
Positive blood culture workflow. Positive blood cultures (+BC) are aliquoted and loaded on the Microbiology Laboratory Automation (MLA) system for Gram stain (GS) slide preparation and subculturing on agar media. Once the prepared slide is GS and read, an aliquot of the +BC is tested using a rapid blood culture identification (BCID) panel based on Gram stain morphology. *BCID is only performed on the first positive blood culture for each patient or if the patient present with a new bloodstream infection (BSI) after resolution of the initial BSI. On the MLA, plates are incubated for up to 24 h with the first image captured at 4 h. If enough growth is detected on the 4 h image, the plates are removed from the incubator for AST and ID by MALDI-TOF MS if the ID was not already available from the BCID. The median TAT for ID by MALDI-TOF MS is ~2:45 h (range: 41 min–8:30 h) and 23 h for AST (range: 16–32 h). AST, antimicrobial susceptibility testing; MALDI-TOF MS, matrix-assisted laser desorption ionization-time of Ffight mass spectrometry; ID, identification.

Blood culture (BC) bottles were incubated in the BD BACTEC FX instrument for up to 5 days. Positive BC bottles were aliquoted in 12 × 80 mm vacutainer tubes (Copan Diagnostics Inc, Murrieta, CA) and loaded on the WASPLab MLA system (Copan Diagnostics Inc, Murrieta, CA) to prepare the Gram stain slides and subculture to bacterial media per standard laboratory protocols. All subcultures were incubated at 37^∘^C and 5% CO_2_. Once loaded on the WASPLab, BC subcultures were initially imaged at 4, 8 16, and 24 h (Fig. 1). When reviewing available images, the technologist recorded the following actions: (i) no growth, (ii) insufficient growth, (iii) same as other bottle, (iv) same as previous recording time, (v) send to positive blood stacker, (vi) culture complete, (vii) contaminant, and (viii) no contaminant. Actions 1 and 2 resulted in further incubation of the cultures, actions 3–4 resulted in no further workup, actions 5 resulted in the culture being removed from the WASPLab for MALDI-TOF MS and AST, action 6 resulted in cultures being finalized, actions 7 and 8 are quality control checks for possible contamination.

### Organism identification

Organism identification was performed from positive BC using the ePlex BCID panels (GenMark Diagnostics/Roche Diagnostics) including the BCID Gram-Negative (BCID-GN) panel and the BCID Gram-Positive (BCID-GP) panel. The BCID panels were selected based on the morphology of the organism observed on the Gram stain of the positive BC. The BCID-GN panel includes the following bacterial targets: *Acinetobacter baumannii, Bacteroides fragilis, Citrobacter* species*, Cronobacter sakazakii, Enterobacter* species (non-cloacae complex)*, Enterobacter cloacae* complex*, Escherichia coli, Fusobacterium nucleatum, F. necrophorum, Haemophilus influenzae, Klebsiella oxytoca, K. pneumoniae* group*, Morganella morganii, Neisseria meningitidis, Proteus* species*, Proteus mirabilis, Pseudomonas aeruginosa, Salmonella* species*, Serratia* species*, S. marcescens,* and *Stenotrophomonas maltophilia*. In addition to the bacterial targets, the following resistance genes are also included in the panel: CTX-M, IMP, KPC, NDM, OXA (OXA-23 and OXA-48), and VIM. Additionally, two Pan targets are included for GN and *Candida*. The BCID-GP includes the following bacterial targets: *Bacillus cereus* group, *Bacillus subtilis* group*, Corynebacterium* species, *Cutibacterium acnes, Enterococcus* species*, E. faecalis, E. faecium, Lactobacillus* species*, Listeria* species*, L. monocytogenes, Micrococcus* species, *Streptococcus* species*, S. agalactiae, S. pneumoniae, S. anginosus* group*, S. pyogenes, Staphylococcus* species*, S. aureus, S. epidermidis, S. lugdunensis*. Resistance genes included: *mecA, mecC, vanA,* and *vanB*. As with the BCID-GN, the BCID-GP includes two Pan targets: Pan GN and Pan *Candida*.

BCID panels were only used on the initial positive blood culture bottle for each patient or on blood culture bottle representing a new BSI episode (i.e., 3 days of subsequent negative BC or no BC received). For subsequent positive blood cultures with similar Gram stain morphology or for cultures with initial negative BCID panels, the identification of the organisms was performed on bacterial colonies growing on solid media from the BC subculture using MALDI-TOF MS (Vitek MS, MYLA v3.0; bioMérieux, Marcy-l’Etoile, France). The blood culture workflow is summarized in Fig. 1.

### Antimicrobial susceptibility testing

AST was performed using the NM53 (Gram-negative), PM34 (Gram-positive), and the Mstrp + 1 (for *Streptococcus* species) microbroth dilution (MBD) panels on the MicroScan WalkAway system (Beckman Coulter, Brea, CA). AST was only performed on one positive BC per day if the organism morphology was identical for all positive BC. Routine AST was not performed for certain organisms (e.g., Gram-positive rods or anaerobic organisms) per the laboratory workflow.

### Data analysis

Data on all actions taken for the 4 h image reading time were retrieved from the WASPLab WebApp. Additional data analysis focused on cultures removed from the MLA for workup (i.e., action #5). Information on organisms reported, the method of identification used (i.e., BCID or MALDI-TOF MS), and the time to MALDI-TOF MS ID and AST results was extracted from the Laboratory Information System (LIS) (Cerner Millennium). Culture results were reviewed to compare results obtained from the early MALDI-TOF MS ID to the IDs reported in other cultures (e.g., ID from follow-up cultures). AST results from the early growth were compared to AST results from standard 16–24 h growth (e.g., AST from follow-up cultures). Only AST results reported were analyzed to determine agreement. For GN, the following antimicrobials were analyzed: amikacin, ampicillin/sulbactam, ampicillin, aztreonam, cefazolin, cefepime, ceftazidime, ceftriaxone, ciprofloxacin, ertapenem, gentamicin, imipenem, levofloxacin, meropenem, piperacillin/tazobactam, tetracycline, tobramycin, and trimethoprim/sulfamethoxazole. For GP, the following antimicrobials were analyzed: ampicillin, penicillin, clindamycin, ceftaroline, oxacillin, ceftriaxone, tetracycline, trimethoprim-sulfamethoxazole, vancomycin, daptomycin, linezolid, levofloxacin, erythromycin. Categorical agreement (CA), minor errors (mE), major errors (ME), or very major errors (VME) were calculated using standard AST (i.e., from 16 to 24 h growth) as the reference method. Discrepant results were investigated by performing repeat testing of the frozen bacterial stock of the isolate initially recovered at 4 h. Frozen isolates were sub-cultured twice before performing AST and 16–24 h growth was used to repeat AST (24 HR). Repeat results were compared to the early growth AST (i.e., 4 h: 4H) and the standard growth AST (i.e., 16–24 h; 24H).

## RESULTS

**Fig 2:**
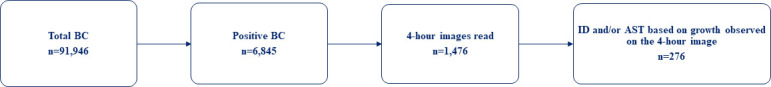
Flowchart of blood cultures with subcultures worked-up based on the review of the 4 h images. A total of 276 cultures (4%: 276/6,845) were worked up for AST and/or MALDI-TOF MS based on review of the 4 h images. BC, blood culture; ID, identification; MALDI-TOF MS, matrix-assisted laser desorption-ionization time of flight. BCID, blood culture identification molecular panel.

A total of 6,845 BC flagged positive during the study period (Fig. 2): 1,476/6,845 (21.6%) 4 h images were reviewed, and 276/6,845 (4.0%) images were deemed to have sufficient growth for cultures to be worked up for ID and/or AST (action #5) (Example in Fig. S1). Organism identification was already obtained by BCID for 124/276 BC (44.9%), and therefore, no MALDI-TOF MS was performed. BCID identified a total of 136 organisms including 9 polymicrobial cultures. A total of 97 Gram-negative (GN) and 39 Gram-positive (GP) bacteria were identified. The most common organism identified was *K. pneumoniae* (*n* = 49) for GN bacteria and *S. aureus* (*n* = 9) for GP bacteria (Table 1). For the other 152/276 cultures (55.1%) without BCID results, MALDI-TOF MS was performed to identify the bacterial organism from the 4 h growth. A total of 153 organisms were identified: 106 GN and 47 GP bacteria. Like the BCID identifications, the most common GN identified by MALDI-TOF MS was *K. pneumoniae* (*n* = 63) and for GPs, *S. aureus* (*n* = 25) (Table 1).

**TABLE 1:** Organisms identified from growth detected at the 4 h MLA digital image

	Organism ID	ID by BCID	ID by MALDI (BCID negative)^*a*^	ID by MALDI (subsequent culture)^*b*^
Gram positive	*Bacillus cereus* group	3	1	1
	*Enterococcus faecium*	6	–^*f*^	8
	*Enterococcus faecalis*	2	–	3
	*Staphylococcus species*	2	–	–
	*Staphylococcus epidermidis*	2	–	4
	*Staphylococcus aureus*	9	–	25
	*Streptococcus bovis* group^*c*^	1	–	1
	*Streptococcus mitis* group^*c*^	4	–	1
	*Streptococcus pneumoniae*	3	–	3
	*Streptococcus anginosus*	2	–	–
	*Streptococcus salivarus* group^*c*^	4	–	–
	*Streptococcus agalactiae*	1	–	–
Gram negative	*Citrobacter freundii*	2	–	–
	*Citrobacter koseri^c^*	1	–	2
	*Enterobacter cloacae* complex	4	–	4
	*Enterobacter non-cloacae*	1	–	1^*e*^
	*Escherichia coli*	38	3	28
	*Klebsiella pneumoniae*	49	3	60
	*Klebsiella oxytoca*	2	–	1
	*Acinetobacter nosocomialis^d^*	–	1	–
	*Pasteurella multocida^d^*	–	1	–
	*Raoultella ornithinolytica^d^*	–	1	–
**Total**		136	11	142

^*a*^ Included on the BCID panel but not detected or BCID performed but organisms not included in panel.

^*b*^ Organism identified on subsequent cultures from the same episode with first positive culture identified by BCID.

^*c*^ ID to genus level on BCID, species level ID obtained by MALDI-TOF MS.

^*d*^ Organism not included in BCID.

^*e*^ Identified by MALDI as *Pluralibacter (Enterobacter) gergoviae*.

^*f*^ –, no organism ID by the method.

When comparing MALDI-TOF MS and BCID ID, there were four organisms not included in the BCID panel that were ID by MALDI-TOF MS: *Acinetobacter nocosomalis, Pasteurella multocida, Pantoea agglomerans,* and *Raoultella ornithinolytica*. An additional seven organisms that should have been identified by the BCID were only identified by MALDI-TOF MS from the 4 h growth, possibly due to either individual target sensitivity, single-nucleotide polymorphism, or limited strain inclusivity. One organism was identified as *Pluralibacter gergoviae* by MALDI-TOF MS but *Enterobacter* (non-cloacae) species by BCID. *P. gergoviae* was formerly known as *E. gergoviae*. Thus, MALDI-TOF MS provided further speciation but not a new actionable identification (Table 1). For the organisms ID by MALDI-TOF MS, the median turnaround time (TAT) to charted ID result after unloading the plates from the TLA was 2:44 h (range: 41 min–8.5 h).

AST was performed on 160/289 (55.3%) of organisms identified by BCID (*n* = 100) and MALDI-TOF MS (*n* = 60) based on the 4 h growth. AST was not performed on the remaining cultures for the following reasons: insufficient growth (*n* = 36), susceptibilities referred to another culture (*n* = 88), or AST not performed in-house (*n* = 5) (Table 2). Isolates from polymicrobial cultures were set up for AST if bacterial morphologies were clearly distinguishable (e.g., Gram negative vs Gram positive). MALDI-TOF MS was used to confirm each colony’s identification from the purity plate prior to releasing AST results. The median TAT to AST results being charted from the time plates were unloaded from the MLA was 23 h (range: 16–32 h).

**TABLE 2:** Antimicrobial susceptibility testing (AST) performed on organisms identified from growth detected at the 4 h MLA digital image^*a*^

AST set-up	Identified by BCID	Identified by MALDI-TOF MS	Total
Sufficient growth at 4 h: AST performed	100	60	160
Insufficient growth at 4 h: culture re-incubated	33	3	36
Referred to another, same day culture: AST not performed	–^*b*^	88	88
AST not performed in-house	3	2	5
**Total organisms**	136	153	289

^*a*^ MLA, microbiology laboratory automation; BCID, blood culture identification panel; MALDI-TOF MS, matrix-assisted laser desorption ionization time of flight mass spectrometry.

^*b*^ –, no organism in this category.

A subset of patients with cultures initially worked-up from the 4 h growth (*n* = 74) had additional positive cultures from the same day with AST performed using the conventional, 16–24 h subculture growth, allowing for comparison of AST results between early and traditional growth. For GN bacteria, the CA was 98% with 1.6% mE, 0.1% ME, and 1% VME and for GP bacteria, CA was 100% with no errors. Overall CA was 98% with 1.4% mE, 0.1% MEs, and 0.8% VMEs observed (Table 3). One major and one very major error were detected in *E. cloacae* (ceftriaxone) and *E. coli* (ceftazidime), respectively. Discordant AST results are summarized in Table 4.

**TABLE 3:** Comparison of antimicrobial susceptibility testing (AST) results from early growth vs conventional subculture^*b*^

Organism type	Interpretations^*a*^			CA	Error rates (%)		
	S	I	R		mE	ME	VME
Gram negative	706	13	77	782/796 (98.3%)	13/796 (1.6%)	1/706 (0.1%)	1/77 (1%)
Gram positive	95	–	41	136/136 (100%)	–	–	–
Total	801	13	118	918/932 (98.5%)	13/932 (1.4%)	1/801 (0.1%)	1/118 (0.8%)

^*a*^ Interpretations obtained from MICs of isolates set up for AST from the early subcultures.

^*b*^ S, susceptible; I, intermediate; R, resistant; CA, categorical agreement; mE, minor errors; ME, major errors; VME, very major errors.

**TABLE 4:** Summary of discordant antimicrobial susceptibility testing (AST) results^*a*^

Organism	Ampicillin /sulbactam			Cefazolin			Ceftazidime			Ceftriaxone			Ciprofloxacin			Imipenem			Piperacillin /tazobactam			Tobramycin		
	4H	24H	24HR	4H	24H	24HR	4H	24H	24HR	4H	24H	24HR	4H	24H	24HR	4H	24H	24HR	4H	24H	24HR	4H	24H	24HR
*C. freundii*																			I	S	S			
*E. cloacae*							S	I	S	S	R	S							S	I	S			
*E. cloacae*																			I	R	R			
*E. coli*	I	R	R				R	S	R															
*E. coli*	R	I	I																					
*E. coli*																						R	I	R
*K. oxytoca*	S	I	I																					
*K. pneumoniae*				S	I	R																		
*K. pneumoniae*													I	R	R									
*K. pneumoniae*																S	I	I						
*K. pneumoniae*	I	S	S																					
*K. pneumoniae*													R	I	I									

^*a*^ S, susceptible; I, intermediate; R, resistant; 4H, AST set up from the early growth; 24H, other culture from same day/same episode with AST performed from overnight (16–24 h) growth; 24HR, repeat testing using overnight (16–24 h) growth of the isolate initially tested at 4 h.

A total of 12 isolates had discordant results (15 errors: 13 mEs, 1 ME, and 1 VME) between AST results obtained from the early, 4 h growth (4H) and AST from isolates tested from standard, 16–24 h growth (24H). Repeat testing of stored isolates, initially recovered from the 4 h growth, was performed using standard, overnight growth (24 HR) to resolve the discrepancies. Results are summarized in Table 4. Repeat testing results showed agreement with the 4H early AST result for 5/15 discordant results, and agreement with the standard 24H AST result for 10/15 discordant results. The initial VME observed for ceftriaxone on a *E. cloacae* remained a VME.

The BCID panels detected resistance markers mecA (*n* = 3) and CTX-M (*n* = 9). There was 100% concordance between the genotypic resistance markers and the phenotypic AST results from the 4 h growth.

## DISCUSSION

In this study, the utility of reviewing an early time point image, obtained after 4 h of incubation of positive blood cultures subcultures on a MLA system, was investigated. The value of this early time point was assessed based on the percentage of cultures that were worked-up resulting in new identification by MALDI-TOF MS and valid and accurate AST results. While the 4 h time point provided accurate early identification and AST results, the overall number of cultures that were worked-up was minimal at less than 5% of all positive BC.

First, less than 25% of all the available 4 h images were reviewed by the laboratory technologists. This was due primarily to misalignment between the current laboratory workflow and the staffing model. The review of the 4 h images and the work-up of cultures are scheduled exclusively during the day shift (8 am–4 pm) at our institution. Therefore, while a 4 h image might have been available for review between 4 pm and 8 am, there were no technologists available to perform additional testing. Based on the data presented in this study, if the laboratory was staffed 24/7, and 100% of the 4 h images were reviewed, it could be extrapolated that 1,280 cultures (18.7%) instead of 276 cultures (4%) would have been worked-up. However, this analysis has several caveats (e.g., does not account for real-time staffing issues like sick calls or the impact of different pathogens). With the current and well-documented laboratory staff shortages (9, 10), a 24/7 staffing model to take full advantage of this early time point is expected to remain a challenge. However, the data in this study highlights the potential that an early time point, imaged with a MLA system, might have to improve time to diagnosis.

Bacterial biomass from early incubation has been previously shown to be sufficient for both MALDI-TOF MS identification and AST results (8, 11, 12). In one study, identification by MALDI-TOF MS after 4 and 6 h incubation of subcultures from spiked blood cultures was successful in 73% and 85% of cases, respectively (8). In a similar approach, Mutters et al. study reported that for cultures spiked with high concentration of organisms, reliable ID could be obtained after average of 7.9 h of incubation and an overall success identification rate of 98.4% (11). Using patient blood cultures, another group showed an identification rate of 92% from 4 to 6 h subcultures (12). In this current study, MALDI-TOF MS ID from early subcultures resulted in 100% identification rate with high concordance with BCID results. While the accuracy of the MALDI-TOF MS ID from the early subculture was high, the added value following initial use of the BCID panels was limited, with less than 10% of pathogen identification not already provided by the BCID. However, for laboratories not using BCID panels or using them for only a subset of organisms (e.g., only Gram-negative bacilli), the data presented in this study reinforces the value of using early growth for ID as the median TAT to ID by the MALDI-TOF MS being reported was about 2 h and 45 min. Compared to the standard blood culture workflow, which usually includes a 16–24 h incubation, the workflow presented here provides organism ID in less than 7 h from the positive blood culture.

In addition to early organism identification, and more importantly, early, and accurate AST results are necessary to optimize patient therapy. Cherkaoui et al. demonstrated that at 4 h incubation there was enough biomass for 0.5 McFarland used for AST by disk diffusion (DD) for *Enterobacterales* and non-fermenting Gram-negative bacilli although the accuracy of these results was not compared to a reference method (8). Fitzgerald et al. confirmed that AST by DD is reliable with 100% agreements for Gram-positive organisms and 92.9% for Gram-negative organisms (12). These findings align to the current study for which an overall categorical agreement of 98% was observed for MBD results from the early growth. One VME was observed and remained upon repeat testing. This could be due to the presence of heteroresistance or different susceptibility profile for isolates selected for AST at 4 h vs 24 h. Polymicrobial cultures with organisms that cannot be distinguished by Gram stain could also lead to mixed AST results, a risk mitigated in this study with the use of the BCID panels. Polymicrobial cultures would be excluded from early AST unless the organism’s morphologies were clearly distinguishable (e.g., Gram positive vs Gram negative). AST results were reported a median of 23 h after the plates were unloaded from the MLA, which is significantly shorter than the standard workflow that often takes about 48 h or more to complete. These early AST results likely had significant impact on patient management. Future studies comparing the TAT for other image time points (i.e., 8, 16, and 24 h) to the 4 h images are needed to establish the clinical impact of early ID and AST.

This study has some limitations. First, as a single-center study, the results of this assessment might not extend to laboratories with laboratory workflows different from our institution. However, the limitations of the staffing model in our study should provide guidance on the potential value of the 4 h time point for other laboratories using or planning to use a MLA system. Second, given its retrospective design, parallel testing was not performed on all cultures, further reducing the overall numbers of cultures analyzed. There were no carbapenem-resistant *Enterobacterales* (CRE) recovered during the study period, which limited assessment of the accuracy of the early subculture for these significant pathogens. Other resistant organisms (e.g., MRSA, ESBL) were identified, and results were concordant between the BCID genotypic results and the early-growth phenotypic results, suggesting that concordance for CRE might also be expected. Finally, the number of isolates that received AST based on the 4 h growth was small. Further studies with a higher number of isolates for AST comparison will need to be performed to confirm the results obtained in this study.

In summary, this study demonstrates that a 4 h reading time on the MLA system provided early assessment of confluent growth from subcultures of positive blood cultures and that accurate identification and MBD results can be achieved. Implementation and optimization of MLA systems with laboratory workflows should provide opportunity for early targeted antimicrobial therapy and better patient care.

## SUPPLEMENTAL MATERIAL

Figure S1 jcm.01320-24-s0001.docx Example of a 4-hour image blood agar plate with sufficient growth for identification by MALDI-TOF MS and/or AST.

ASM does not own the copyrights to Supplemental Material that may be linked to, or accessed through, an article. The authors have granted ASM a non-exclusive, world-wide license to publish the Supplemental Material files. Please contact the corresponding author directly for reuse.
