# Supplementary material for: Utility of digital images captured after 4 h of incubation on a microbiology laboratory automation system in guiding the work-up of subcultures from positive blood cultures
Source: J Clin Microbiol. 2024 Dec 20;63(2):e01320-24. doi: 10.1128/jcm.01320-24 (PMC11837544; doi:10.1128/jcm.01320-24)
Supplement: Figure S1 — Example of a 4-hour image blood agar plate with sufficient growth for identification by MALDI-TOF MS and/or AST. [file jcm.01320-24-s0001.docx]

Utility of Digital Images Captured After 4-hour of Incubation on A Microbiology Laboratory Automation System in Guiding the Work-Up of Subcultures from Positive Blood Cultures


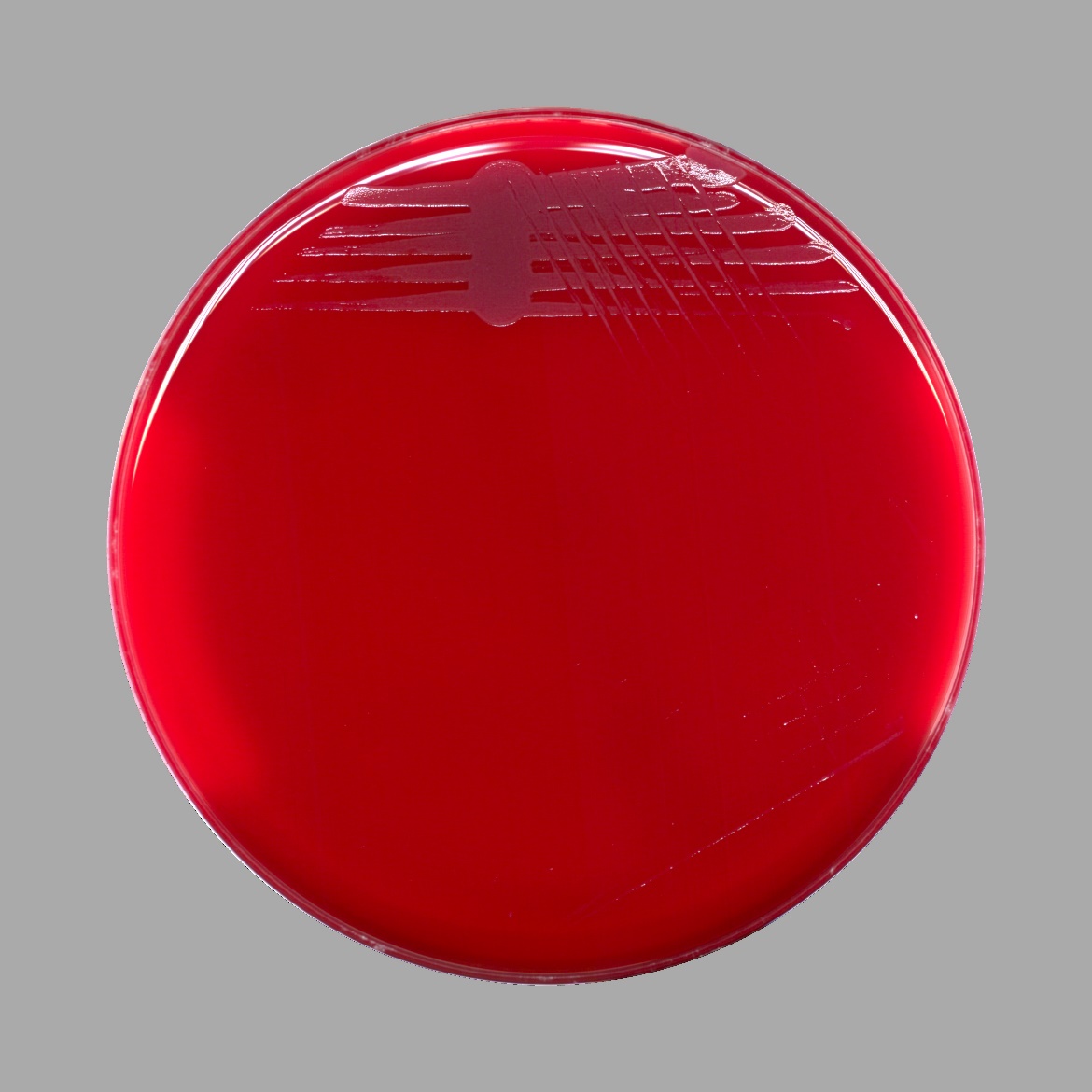


**Supplemental Figure 1.** Example of a 4-hour image Blood Agar Plate with sufficient growth for identification by MALDI-TOF MS and /or AST.
